# A call for a round robin study of XAFS stability and platform dependence at synchrotron beamlines on well defined samples

**DOI:** 10.1107/S1600577518003752

**Published:** 2018-05-29

**Authors:** Christopher T. Chantler, Bruce A. Bunker, Hitoshi Abe, Masao Kimura, Matthew Newville, Edmund Welter

**Affiliations:** a School of Physics, The University of Melbourne, Melbourne, Australia; b Department of Civil and Environmental Engineering and Earth Sciences, University of Notre Dame, IN, USA; c Institute of Materials Structure Science, High Energy Accelerator Research Organization (KEK), 1-1 Oho, Tsukuba, Ibaraki 305-0801, Japan; d Department of Materials Structure Science, School of High Energy Accelerator Science, SOKENDAI (The Graduate University for Advanced Studies), 1-1 Oho, Tsukuba, Ibaraki 305-0801, Japan; e CARS, University of Chicago, Chicago, IL, USA; f DESY Photon Source, Hamburg, Germany

**Keywords:** X-ray absorption fine structure, XAFS, round robin, benchmarking

## Abstract

Round robin studies have been used across fields of science for quality control testing and to investigate laboratory dependencies and cross-platform inconsistencies as well as to drive forward the improvement of understanding of experimental systems, systematic effects and theoretical limitations. Here, following the Q2XAFS Workshop and Satellite to IUCr Congress 2017 on ‘Data Acquisition, Treatment, Storage – quality assurance in XAFS spectroscopy’, a mechanism is suggested for a suitable study across XAFS (X-ray absorption fine-structure) beamlines and facilities, to enable each beamline to cross-calibrate, provide representative test data, and to enable collaborative cross-facility activities to be more productive.

## Abstract 2

A program and methodology are proposed to study X-ray absorption fine structure of potential reference samples across XAS beamlines and synchrotron facilities, to enable each beamline to cross-calibrate, provide representative benchmarks and to enable more productive collaborative cross-facility activities.

## 1. Introduction: round robin tests and the IUCr X-ray attenuation project   

Round robin tests are an established tool to promote interlaboratory comparability of the results of analytical methods. They are usually performed under the auspices of a national or international organization like the IUPAC. The basic idea is that identical samples are analysed by different laboratories using either their own or standardized analytical protocols. Considering that today extended X-ray absorption fine-structure (EXAFS) and X-ray absorption near-edge (XANES) spectroscopy are well established analytical techniques which find widespread applications in many fields of science, it is time to start an investigation into the degree of comparability of XAFS (X-ray absorption fine-structure) results from sources all over the world, even more so since EXAFS/XANES are more and more often used as part of industrial or industry-related research projects. An example from X-ray spectroscopy that can serve as a guideline and underlines the importance of such an attempt is the International Union of Crystallography (IUCr) X-ray Attenuation Project, which was inaugurated by the IUCr Commission on Crystallographic Apparatus in 1979 as a response to widespread discontent among users of X-ray attenuation coefficients with the quality of data available and their inconsistencies. Its main aim was the determination of preferred experimental techniques for the measurement of X-ray attenuation coefficients. A secondary aim was the evaluation of theoretical and semi-empirical compilations (Cromer & Mann, 1968 ▸; Cromer & Liberman, 1970*a* ▸,*b* ▸, 1981 ▸; Storm & Israel, 1970 ▸; Scofield, 1973 ▸; Hubbell *et al.*, 1975 ▸; Saloman *et al.*, 1988 ▸).

That round robin was associated with attenuation, or perhaps absorption, and not X-ray absorption fine structure or X-ray fluorescence fine structure. In other words, the structure above absorption edges was not a direct concern. That extensive study took more than ten years to complete and four years to write up the results. The sources compared corresponded to the major facilities of the period – that is, they were laboratory sources of differing complexity, rather than synchrotron sources. However, the work of that group and volunteers across the world led to an understanding of some of the key experimental needs of the field at that time, some of the lessons from which remain valid for absorption and hence XAFS today.

One key theoretical outcome of that study was to conclude that the coherence of the elastic scattering processes could dramatically affect the measurements and in an energy and orientation-dependent manner depending upon whether the interaction was characterized by Rayleigh, Bragg–Laue or thermal diffuse coherent elastic scattering. A second conclusion was that the Scofield tables (Scofield, 1973 ▸) with or without correction for the relativistic nuclear amplitude were a useful resource, leading to uncertainties corresponding to the magnitude between these two values and leading to the development of the first NIST reference database for attenuation (Saloman *et al.*, 1988 ▸; Berger *et al.*, 1999 ▸).

Further inconsistencies in available theoretical sources of atomic form factors and attenuation databases led to the development of relativistic codes and corrections using density functional theory (DFT) and Kohn–Sham potentials (Brennan & Cowan, 1992 ▸; Creagh & Hubbell, 1992 ▸; Creagh & McAuley, 1992 ▸; Chantler, 1993 ▸, 1994 ▸; Bergstrom Jr *et al.*, 1997 ▸) and new theoretical standards and the NIST reference database for attenuation, form factors and atomic scattering tables FFAST (Chantler, 1995 ▸, 2000 ▸). Meanwhile, compilations of attenuation or absorption coefficients from the literature began to be developed (Henke *et al.*, 1982 ▸, 1993 ▸; Hubbell, 1994 ▸, 1999*a* ▸,*b* ▸, 2003 ▸; Cullen *et al.*, 1997 ▸). These continued to concentrate on absorption, attenuation and form factors rather than the edge structure, both because the additional processes involved divergence from one-electron or atomic approximations and due to the extreme difficulty in presenting detailed fine structure in a compact or printed tabulation. Indeed, the major discrepancies between the two NIST reference databases (Chantler, 2000 ▸; Saloman *et al.*, 1988 ▸) lie unsurprisingly in the region of the absorption edges both because of the complex interference structure, the difficulty of defining the absorption edge energies purely theoretically, and the limitations of DFT in general.

Some key possible purposes of a round robin activity are:

(i) The round robin can comment on and establish to which degree XAS data can be cross-compared. In particular, the round robin could establish the general framework under which data may be cross-comparable.

(ii) The round robin can be a seeding activity to generate a database of re-usable XAS data.

(iii) The round robin can tell us what the key incomparable differences are between different types of beamlines. This could be input information into a framework for data sharing.

(iv) The round robin can be an initial activity for a practical XAS standard approach.

## 2. Preliminary experimental progress and lessons   

The first series of tests in the round robin on attenuation coefficients (Creagh & Hubbell, 1987 ▸; Mika *et al.*, 1985 ▸) were made on the attenuation of (single-crystal) crystalline silicon across the energy range 8–60 keV, hence posing a challenge with Bragg–Laue diffraction peaks and the general form of elastic scattering. The materials in this attenuation project were chosen because they posed well defined experimental problems which would test both experimental equipment and experimental technique. Two sets of silicon specimens were prepared. One set was cut from a cylindrical boule of high-purity electron-beam float-zoned single-crystal silicon, the cylinder axis of which lay parallel to [220]. The other set was cut from a cylindrical boule of similar purity but which had the cylinder axis within 0.25° of parallel to [111]. The density of the samples was measured. The samples had a surface area of 15 mm × 15 mm and thickness varying from 0.4 mm to 4 mm, chosen to enable the Nordfors criterion (Nordfors, 1960 ▸) for optimum counting statistics to be fulfilled by either one specimen or a combination of specimens for wavelengths commonly used by crystallographers. Seven samples were distributed to each laboratory.

Samples were sent to 25 laboratories worldwide. Participants in the project were asked to select whatever technique of measurement they thought appropriate for the specimen and photon energy selected for that measurement. Twelve laboratories reported with results using a total of eight different experimental configurations. Most laboratories measured coefficients for three characteristic energies (Cu *K*α_1_, Mo *K*α_1_, Ag *K*α_1_). Most experiments were performed with conventional X-ray sources using characteristic radiation whilst others were performed using bremsstrahlung radiation; two different X-ray fluorescence configurations were used and emissions from the radioactive source ^241^Am were used by two laboratories. Most experiments used some form of energy discrimination

For this purpose the study found closest agreement with a model for scattering following thermal diffuse rather than Rayleigh scattering. Three key experimental concerns raised in this study were the minimization of second-harmonic contamination, the use of a dead-time correction, and the reduction of any large divergence. The latter indicated the need for a monochromator.

The second series of tests related to pyrolytic graphite, known to contain a relatively high density of voids, which would produce significant small-angle X-ray scatter, and also with no *K*-edge in the region of interest (Creagh & Hubbell, 1990 ▸). The samples were taken from a stock of POCO graphite donated by Poco Graphite Inc., Texas, USA. Sheets of POCO graphite ranging in thickness from 0.127 to 1.016 mm were cut into 15 mm squares. Each participant in the project was given about ten different thicknesses of sample. A key part of this project was the measurement of the density of the specimens by participating laboratories. For carbon only five experimental configurations were used. The principal conclusion of their survey of measurement techniques was that a variety of poorly understood and unquantified sources of systematic error may be adversely affecting the measurements. The data were consistent to better than 5% for the energy range 6 to 60 keV, but were about 3% less than that given by Saloman *et al.* (1988 ▸). Here eight characteristic energies were used for measurement.

The third series of tests related to highly rolled polycrystalline copper metal foils, which might produce significant scattering because of preferred orientation within the foils caused by the rolling process (Creagh, 1987 ▸). A key observation here was that such copper samples provided elastic scattering best represented by Rayleigh (incoherent) scattering rather than any collective effects such as Bragg–Laue or thermal diffuse scattering.

Even for attenuation and absorption, it has been realized that synchrotrons offer similar advantages to those for XAFS, so high-accuracy absorption experiments have turned increasingly to synchrotron sources (Hubbell *et al.*, 2003 ▸; Chantler *et al.*, 1999 ▸, 2001*a* ▸; Tran *et al.*, 2003*a* ▸,*b* ▸). Initially the variability of the source position, size, energy and flux were considered to be likely to yield lower accuracy than a stable laboratory or national laboratory source, but the capacity with the high fluxes to address and constrain these and other possible systematics has generally led to higher quality datasets.

## 3. Background: IUCr and IXS standards and criteria   

A related initiative working towards a round robin commenced both from the IUCr and the then young International X-ray Absorption Society (IXAS, previously IXS). This led to several meetings (Lytle *et al.*, 1989 ▸), a workshop and a report (Sayers, 2000*a* ▸,*b* ▸) over a similar time period, however, without a resulting laboratory or synchrotron benchmark or round-robin. XAFS began with local sources (Fricke, 1920 ▸; Lytle, 1965 ▸, 1966 ▸; Lytle *et al.*, 1975 ▸), but it was soon understood that synchrotron sources dominated for the acquisition of high-flux high-precision data sets (Lapeyre *et al.*, 1983 ▸; Doniach *et al.*, 1997 ▸; Lynch, 1997 ▸). Indirectly, this initiative has led to the development of some standard practices at synchrotron beamlines for sample preparation and data collection (Newville, 2004 ▸; Bunker, 2010 ▸).

## 4. Past cross-calibration activities   

At XAFS beamlines at major synchrotrons, cross-calibration activities are usually internal to a particular synchrotron and beamline and may check alignment, monochromator calibration, hysteresis, mirror or detector functioning or a wide range of possible systematics or local glitches. The only readily accessible sources for such calibration of XAFS beamlines or monochromation energies have been the Lytle database (http://ixs.iit.edu/database/data/Farrel_Lytle_data), with highly variable content and format, the spectral profiles of Wong (1999 ▸) used as standards by numerous beamlines, but without absolute calibration, and more recently edge definitions (Deslattes *et al.*, 2003 ▸) of variable provenance given unaccounted broadening and instrumental effects. Weaker energy calibrations have used a single edge position using older tabulations.

A cross-calibration exercise was run on several beamlines at the Advanced Photon Source (APS), Chicago, USA, to calibrate performance across XAFS or XAFS-like beamlines. Samples used then included copper metal samples, silver metal samples and a dilute solution cell sample. Perhaps understandably, reports of this exercise, or a definition of a standard profile, did not eventuate, because the purpose was to investigate beamline functionality.

One example of a cross-calibration exercise investigated the correlation of 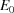 and 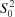 from different beamlines (Kelly, 2009 ▸).

## 5. XAFS standards for a round robin   

More recently, newer experimental XAFS standards include those of Chantler *et al.* (Chantler *et al.*, 2001*a* ▸,*b* ▸; Tran *et al.*, 2005 ▸; de Jonge *et al.*, 2005 ▸, 2007 ▸; Glover *et al.*, 2008 ▸, 2010 ▸; Tantau *et al.*, 2015 ▸; Chantler, 2009 ▸) and Kraft *et al.* (Kraft *et al.*, 1996 ▸), which show some promise of being valuable as cross-calibration standards. Both use a methodology of calibration by a second analyser crystal with multiple lattice planes, so energy can be well calibrated, and in principle a calibration can be established across more than a single point corresponding to a notional edge position or inflection point. Both use synchrotron monochromation as a source, and both use transmission measurements through the sample rather than fluorescence measurements of XAFS. Chantler and Kraft both use variations of the Bond method (energy calibration using positive and negative reflections with an X-ray lattice standard) in their analysis (Bond, 1960 ▸; Deutsch *et al.*, 1995 ▸). Kraft used a narrow bandwidth due to the use of a four-bounce monochromator, but provides only edge positions in the manuscript using the inflection point of lowest energy in the spectrum taken under good conditions. Conversely, the Chantler group have provided detailed XAFS and edge positions for copper, silver, gold, molybdenum, tin and rough XAFS for some other materials, especially in data formats in tables and supplementary information (Chantler *et al.*, 2001*a* ▸, 2015 ▸; Tran *et al.*, 2003*b* ▸, 2005 ▸; de Jonge *et al.*, 2005 ▸, 2007 ▸; Glover *et al.*, 2008 ▸; Islam *et al.*, 2014 ▸, 2016 ▸; Tantau *et al.*, 2015 ▸). More work is doubtless needed in this exercise, but it paves the way for future higher accuracy and standard cross-calibration.

## 6. XAFS studies and preparation for a round robin: recent experimental lessons   

It is important to consider the most accurate and recent efforts to provide stable references for XAFS and XANES and related techniques and not just for absorption or attenuation. More recently, the Chantler group measurements showed the importance of using multiple thicknesses in the determination of 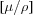, and also of choosing thicknesses well outside the Nordfors criterion 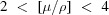, as opposed to the earlier studies. They emphasized the importance of energy calibration using X-ray diffraction peaks, statistical processing of uncertainty and characterization of morphology of samples *via* mapping the integrated column density across the sample, and some absolute calibration of thickness to yield an accurate transfer for 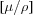. Kraft *et al.* (1996 ▸) likewise emphasized the Bond method (Bond, 1960 ▸; Deutsch *et al.*, 1995 ▸), a very narrow divergence, a narrow bandwidth from a four-bounce monochromator, and the removal of parasitic reflections by detuning one arm of the monolithic monochromator. Interestingly, the Kraft *et al.* (1996 ▸) measurements were made at liquid-nitrogen temperatures, unlike most use of reference foils as standards; most Chantler group measurements have been made at a well defined room temperature. The Kraft *et al.* (1996 ▸) measurements were apparently not sensitive to the thickness ratio relating to the Nordfors criterion (in this case the effect shifting the apparent edge position due to differential absorption from the bandwidth) because the bandwidth of the four-bounce monochromator was extremely narrow. Conversely, the X-ray Extended Range Technique of the Chantler group uses the bandwidth and near-edge thickness effect to measure the bandwidth of the beam and remove the systematic shift. Both groups used multiple measurements to assess statistical uncertainty and other effects. Finally, the Kraft *et al.* (1996 ▸) measurements were designed to define the inflection point 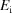 and not the absolute or independent 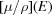. Kraft *et al.* (1996 ▸) do not report the sample characteristics.

Copper metal foils of high quality (Goodfellow) (Chantler *et al.*, 2001*a* ▸,*b* ▸; Glover *et al.*, 2008 ▸) were investigated across the edge but more broadly across the range 8.5−20 keV using samples of thicknesses 5, 10, 15, 20, 30–100 µm, of purity 99.99%, and following the criterion at edge (below) (0.08–0.5) < 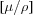 < (1.4–5) (above). Most of these investigations by the Chantler group have used samples which are flat and approximately 25 µm × 25 µm squares, mounted in plastic holders. Silver metal foils of high quality (Goodfellow) were investigated across the edge but more broadly across the ranges 15–50 keV (Tran *et al.*, 2005 ▸) and 11–28 keV (Tantau *et al.*, 2015 ▸; Chantler, 2009 ▸) using samples of thicknesses 5, 10, 12, 50, 100–275 µm, of purity 99.99%, or 1, 10, 12.5, 50, 100 µm and 99.95+%, following the criterion 0.5 < 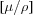 < 5.

Molybdenum metal foils of high quality (ESPI) (de Jonge *et al.*, 2005 ▸) were investigated across the edge but more broadly across the range 13.5–41.5 keV using samples of thicknesses 25, 50, 100, 150, 200, 250 µm, of purity 99.98%, following the criterion (0.3–2) < 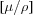 < (3.5–9). Tin foils (ESPI) (de Jonge *et al.*, 2007 ▸) were investigated across the edge but more broadly across the range 29–60 keV using samples of thicknesses 25, 50, 100, 150, 200, 250, 500 µm, of purity 99.99%, following the criterion (0.1–0.9) < 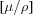 < (2–7.5). Gold foils produced by Goodfellow were investigated across the *L*_I_-edge but more broadly across the range 14–21 keV (Glover *et al.*, 2010 ▸) using samples of thicknesses 5, 9, 15, 25 µm, of purity 99.99% or 99.9%, following the criterion (1–2) < 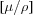 < (3.4–5.2). Other investigations did not particularly concentrate on a detailed mapping of the edges and XAFS but rather defined an absorption and attenuation standard. Silicon and carbon have no absorption edge in the X-ray region accessed by most XAFS beamlines, so, while they can be good tests of attenuation, they are not useful tests of XAFS accuracy or consistency.

Notice that all of these standard measurements used transmission, *i.e.* absorption, to define an absolute or relative attenuation coefficient, rather than using fluorescence XAFS. This raises a tough and unanswered question as to what good technique would be used for fluorescence XAFS. This round robin might provide answers to this question.

## 7. Proposal for benchmarking and call for a round robin   

With the rapid growth of synchrotrons and XAFS-related beamlines, attention has swung to the consistency of data from different beamlines and the cross-portability of data, with respect to data formats, data collection, data content and beamline dependencies. A user may want to know the quality of the data collected on a given day; the beamline scientist will want to rely upon the standard protocols to a known level of accuracy or insight; the management will want to confirm the outstanding results obtained in the laboratories and beamlines under their control. Due to the high monochromatic flux at most XAFS beamlines around the world and hence high statistical precision per data set, the observed variance between spectra measured at different places must be due to factors other than the purely statistical (Chantler *et al.*, 2012 ▸).

A critical mass of experts are now calling to address and improve this situation experimentally and internationally (Ascone *et al.*, 2012 ▸; Chantler *et al.*, 2012 ▸; Diaz-Moreno, 2012 ▸; Ravel *et al.*, 2012 ▸). This has now led to the current proposal and exercise to pursue a round robin evaluation of standard samples. Optimistically, the exercise will lead to more of the advances established by previous round robin and calibration efforts.

Hence we propose to define a specification for all laboratories to measure the *K* absorption edges and XAFS region (XANES and EXAFS) of previously agreed representative samples, calibrated in any way they see fit, and return the resulting data and discussion with any issues of importance relating to the use of the data and spectra for XAFS. They may report using absorption (transmission) or fluorescence, or indeed any variant.

In particular, we currently have support and sign-on from interested parties from the Q2XAFS Workshops, from DESY, PETRAIV (E. Welter, Germany), APS (B. Bunker, M. Newville, USA), Diamond (S. Diaz-Moreno, F. Mosselmans, G. Cibin, UK), ESRF (S. Pascarelli, France), Elettra (G. Aquilanti, Italy), KEK (H. Abe, M. Kimura, Japan), SPring-8 (T. Watanabe, T. Homma, Japan), Stanford (R. Sarangi, A. Mehta, USA), Swiss Light Source (M. Nachtegaal, Switzerland), Sirius LNLS (S. Figueroa, Brazil), MAX IV (K. Klementiev, Sweden), Institute for Photon Science and Synchrotron Radiation, KIT (S. Mangold, Germany), AS (P. Kappen, Australia) and others. This list is not exclusive and interested beamline responsible personnel are welcome to contact us and participate.

Our initial direction is to observe the variability of spectra measured at different beamlines, pre-edge, XANES, XAFS to high-*k* and indeed the variability of reported χ *versus* *k* spectral profiles. Equally, this can lead to variabilities in 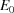, 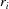 and any XAFS fitting parameters. Having observed spectral variation and structural changes, it will become important to characterize causes and make more reliable recommendations for sample preparation, data collection and analysis to enable more insightful calibration, cross-calibration and experimental outcomes for facilities and users. In particular we would like to investigate: energy scale, scan-to-scan energy variations, energy resolution, and obvious systematic errors and glitches. Obtaining reliable, transferable and robust independent measurements of XANES would be a really good early goal. This will lead to later more insightful investigations of analysis and robustness.

**Figure 1:**
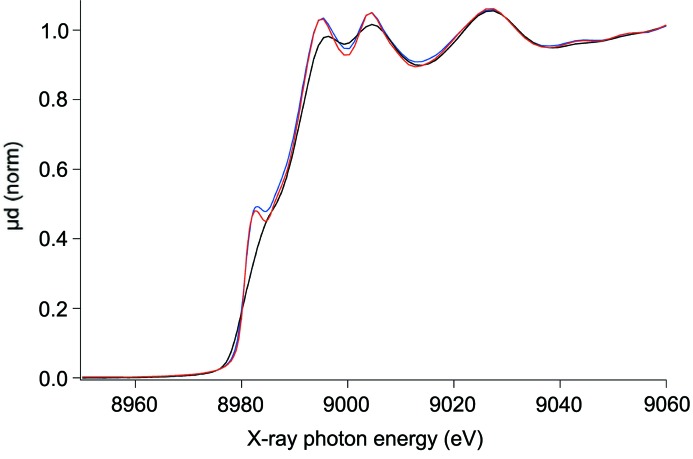
Three copper metal foil spectra, two of which were measured at P65 at PETRA III using different vertical slit sizes (blue, black); the third one is taken from the XAFS spectra library (http://cars.uchicago.edu/xaslib/spectrum/606). All three were measured at room temperature using Si(111) double-crystal monochromators. The comparison with an externally measured spectrum clearly demonstrates that even the blue spectrum is broadened by suboptimal energy resolution.

For this first round robin which is addressing beamline performance and not sample (preparation) related parameters, we propose the following materials:

(i) Copper metal foils, 99.99% pure, multiple thicknesses, 5 µm, 10 µm, 20 µm, 40 µm at room temperature [*K*-edge estimated as 8980.476 (20) eV (Kraft *et al.*, 1996 ▸)]. Copper is the most tested X-ray standard, for the copper metal edge profile and XAFS. It is therefore the primary active standard for most synchrotron sources and calibration activities and has the strongest background of experimental provenance (Fig. 1 ▸).

(ii) Molybdenum metal foils, 99.98% pure, multiple thicknesses, 25 µm, 50 µm, 100 µm [*K*-edge estimated as 20000.351 (20) eV (Kraft *et al.*, 1996 ▸)]. Most synchrotron XAFS beamlines go up to 20 keV and so this energy is a useful higher energy point for cross-calibration purposes. Silver metal foils could also be an excellent standard but the *K*-edge is at 25.5 keV and is not accessible by numerous XAFS beamlines, so we suggest that molybdenum is a better material as a complement to copper metal foils. Tin metal is also a possible tool but has a yet higher edge at 29 keV, beyond the reach of a range of beamlines.

(iii) Titanium metal foils, 99.5+% pure, multiple thicknesses. Titanium is a good standard for lower hard X-ray range XAFS measurements. This represents a reasonable lower energy limit for standard foils. In addition, it is suitable for examining harmonics contamination, whether mitigated by detuning or harmonic rejection mirrors. This is expected to be a more challenging comparison but is at the lower end of the range for non-vacuum beamlines and standard XAFS beamlines and monochromators. It is noteworthy that neither Kraft *et al.* (1996 ▸) nor Deslattes *et al.* (2003 ▸) nor Chantler have a published measurement of the Ti *K*-edge. Bearden & Burr (1967 ▸), uncalibrated, report 4966 eV from Å* which is largely obsolete.

Light-tight samples of standard nominal thickness, *e.g.* from Goodfellow, will be distributed to each facility. Laboratories can use all of these and report one result, or use each and any of them and report individual results. We recommend samples of size 25 mm × 25 mm, held in some mount, as this is a standard source material and suitable for many beamlines. However, some beamlines may have and use standard 10 mm × 10 mm or a size appropriate for their standard mounts. Any cryostat samples and mounts are likely further constrained.

In a recent Japanese effort related to the Japanese Ministry of Education, Culture, Sports, Science and Technology (MEXT) initiative of the ‘Photon beam platform’ (http://photonbeam.jp), foils of Ti, Cu, Zr, Pt and more were used. Zn, Zr, Pt, Sn, Ag and Au remain good candidates for future study and cross-comparison, though the high-energy edges are likely for a smaller number of synchrotrons with appropriate energy coverage.

We expect that these pure metal samples will be able to be measured and characterized more readily using XAFS transmission mode data collection rather than using fluorescence data with significant self-absorption; however, many beamlines are able to measure such samples in both transmission and fluorescence, and for numerous beamlines a fluorescence spectrum will be a ‘standard result’. Hence investigating both will comment on the ease of transfer standards for fluorescence measurement. It is also possible to generate thin metal foils mounted on backing for fluorescence measurement. These are poor standards for transmission because of the backing but can be particularly valuable at soft X-ray energies and for fluorescence samples.

**Figure 2:**
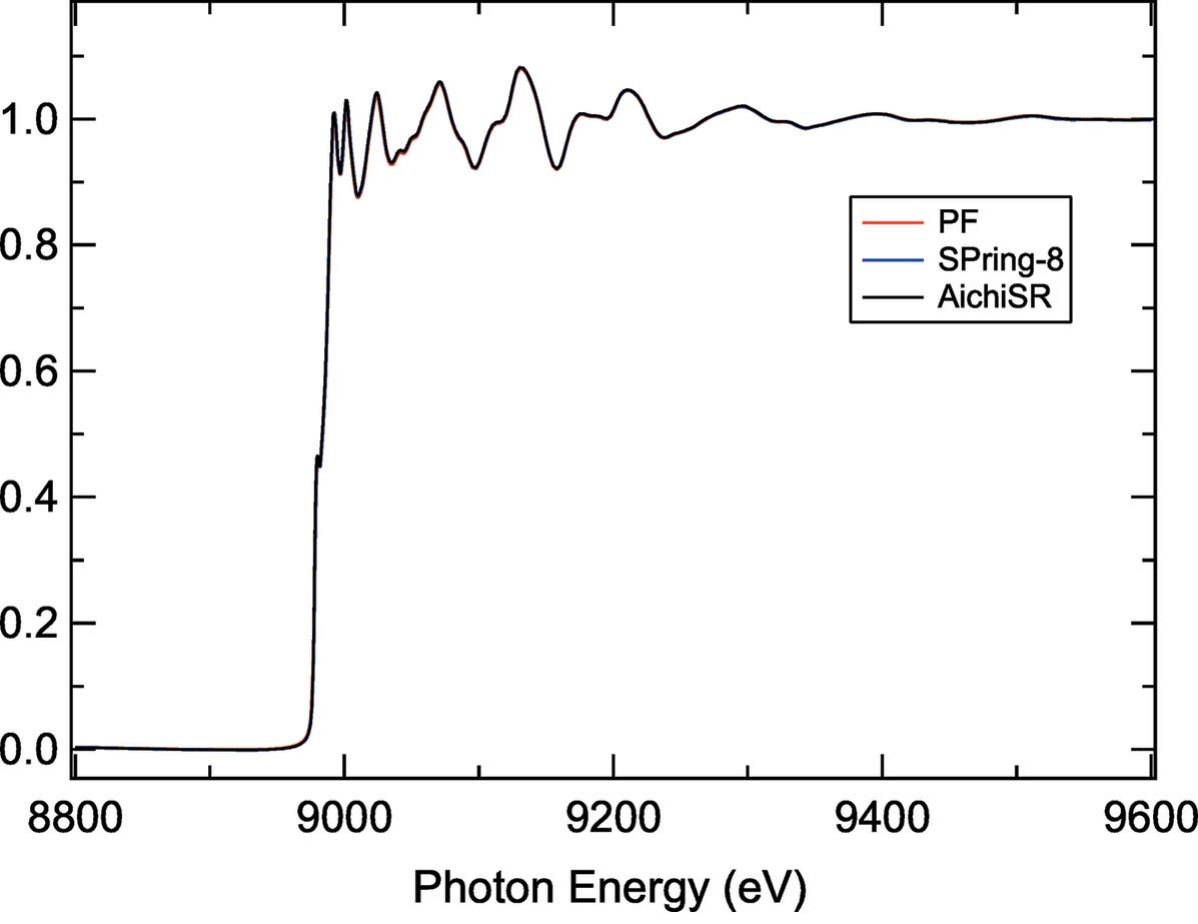
Cu foil XAFS spectra measured at Photon Factory (red), SPring-8 (blue) and AichiSR (black). These data were obtained by the Japanese Round Robin activity (K. Kimijima and M. Masao).

Even for copper metal foil we expect to see significant differences in the XAFS spectrum and perhaps especially in the pre-edge and near-edge region (Figs. 2 ▸ and 3 ▸).

## 8. Second stage: powders and oxides   

After the completion of the first round of round robin tests using pure metal foils a second round will address the most often used types of samples and the specific problems which arise from sample preparation and stability in the photon beam. Most often, XAFS samples are initially powders which are prepared in one or another way into more or less homogeneous samples. These sample preparation methods and the influence of them is of major interest to any user of XAFS spectroscopy. Another class of interesting samples are liquid samples which are common in biological XAFS applications but also in homogeneous catalysis. Here, obviously, homogeneity is not the main concern, but stability in the X-ray beam and, especially for fluorescence-yield XAFS, the huge scattering background produced by the solvent.

The best provenance powders are the NIST reference powder standards, SRM 674b, including TiO_2_, Cr_2_O_3_, ZnO, CeO_2_, which are available as certified reference materials for powder diffraction. We are therefore recommending for the powder trials, firstly, the use of powders with full provenance of size distribution and purity (*i.e.* NIST SRM 674b). Secondly, we recommend that local beamlines that have sourced this material then prepare them in their standard or best practice method, to look towards separating out the preparation method issues from the sample morphology issues in future investigations.

First trials should work with TiO_2_, Cr_2_O_3_ and ZnO, as representative of energy ranges suitable for almost all standard XAFS beamlines. Additionally, the titanium samples will require more care with ion chambers, flight paths and sample preparation method, and lie at the lower end on the hard X-ray energy range; while the chromium and zinc oxides are stable and in a more robust energy range. For high energies and diffraction orders we can recommend CeO_2_. All metal oxides have light backscatter from the first shell and they are sufficiently stable for high accuracy and reliable measurements. Where there is no pre-existing certificate of standard reference sample distribution, the samples should (also) be characterized using another analytical method, *e.g.* X-ray diffraction (XRD), and then provide sub-samples of one well characterized batch to the participating beamlines.

In later trials, we anticipate one or two laboratories producing a set of standard samples for distribution to all interested beamlines (*e.g.* a ground powder of a standard, mixed with boron nitride or cellulose into a compressed pellet of defined diameter; or a ground powder attached to multilayers of ‘magic tape’) and that these two alternate sample standards be distributed for the cross-calibration. Cross-checking of samples prepared in other laboratories would be welcomed.

As an example, the Japanese MEXT initiative is proposing the following recipe for preparation of standard powder pellets:

(i) Weigh sample powder in order to have 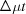 ≃ 1.

(ii) Weigh 70 mg of boron nitride.

(iii) Mix sample powder and boron nitride in a mortar.

(iv) Make a standard pellet with a table press or pellet maker; adjust the amount of sample to the pellet size.

The recent and ongoing Japanese MEXT initiative is also looking at dilute powders rather than concentrated or pure powders. In this context they are looking at TiO_2_ and, for example, 10 parts per million (p.p.m.), 50 p.p.m., 100 p.p.m. of Cu, CuO samples mixed with boron nitride will be measured. In other words, looking at a well ground powder sample of copper metal combined with a similarly prepared sample of CuO would be an excellent cross-calibration activity in the central X-ray energy range and sensitive to statistics and systematics and also sensitive to powder sample preparation. This might also link in directly to the many fluorescent beamline arrangements and permit further cross-calibration between transmission and fluorescence detection measurements.

## 9. Later stages: glasses and dilute solutions   

Glasses or samples mixed in glasses in dilute forms can be ideal for XAFS measurements and cross-calibration if they are characterized well in terms of local structure, clustering and matrix. Once formed (possibly in a batch for distribution) and once characterized (possibly by a variety of methods, *e.g.* XRD, UV/Vis, IR/Raman spectroscopy *etc*.), they can have good homogeneity, good uniformity, control of sample shape and ease of handling. They are popular among X-ray fluorescence users and available as certified reference materials (from NIST *etc*.).

As a first step, we recommend a NIST SRM for cross-calibration; once again, a later step can involve (two) independent laboratories distributing alternate sample preparations to see the uniformity of resulting structure and XAFS interpretation. The SRM 2241 is 0.02% Cr_2_O_3_ in a sodium borosilicate matrix, 10 mm × 10 mm × 1.65 mm, so still far from optimized for transmission but potentially fine for fluorescence measurement of XAFS. Similarly, SRM 2242 is a manganese-doped (0.15 wt% MnO_2_) borate matrix glass. There are two different formats of SRM 2242 to which the certified properties in this certificate apply. One format of the SRM consists of a glass slide that is approximately 10.7 mm in width × 30.4 mm in length × 1.6 mm. This is not well optimized for transmission XAFS measurement but could be quite suitable for fluorescence-yield XAFS measurement. Additionally, or alternatively, one or two laboratories can prepare common glass samples for distribution and analysis in a batch.

Later investigations can involve well characterized dilute solution samples from biological XAFS to address radiation damage, ice crystallization, freezing samples and low concentrations such as for Fe *K*-edge XAFS of haemoglobin in water.

## 10. Methods   

Samples will be sent to participating laboratories wherever possible. In general, the participating laboratories should mount and collect data in whichever method they think best or common. The first samples are foils, concentrated and pure, so we recommend that beamlines start with transmission mode and, later, in a second step, these or other samples may be considered for fluorescence-yield measurements. Suitably optimized samples can be used for both, simultaneously. Each laboratory that participates is expected to follow its own measurement protocols, if necessary pre-processing and annotation of conditions for each relevant beamline, and pass this on to a central source for further processing. Spectra should be submitted as plain ASCII files. A standardized data format such as xdi (Ravel *et al.*, 2012 ▸) is preferable but not mandatory as long as the used data format is well enough documented to use the files without too much additional effort.

Although each laboratory should follow its own protocols, some important parameters with influence on the anticipated results can be *commended* to obtain meaningful scan results for the round robin test:

(i) Length of a scan: as far out as possible. This is an interesting result, in fact we see some differences in TiO_2_ for *k* > 12 Å^−1^ (Japanese Round Robin).

(ii) Resolution/regions file: the resolution (step size in the case of stepwise scans) in the XANES region should meet the spectral resolution of the monochromator used. For the most often used Si(111) that would mean about 0.5 eV in the case of the Cu *K*-edge and about 0.25 eV in the case of the Ti *K*-edge. The step size in the EXAFS region can be set to 0.04 or 0.05 Å^−1^ or equivalent values in energy.

(iii) Scans in continuous or stepwise scan mode are welcome. The submitted data should have the same density of points as routinely used at a beamline, including any form of rebinning if this is already done by the beamline software.

(iv) Temperature: room temperature for the first round using metal foils; low temperature for some later investigations.

(v) Output data formats: 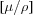 *versus* *E* in ASCII columns of text with header and annotation (*e.g.* such as for xdi). Also, a processed χ *versus* *k* is expected. Other additional formats and data are optional.

Ideally the resulting spectra are stored in one or more XAFS spectra repositories (see other contributions of this issue) and will be available there for further evaluation and as reference spectra for internal beamline testing at existing and new XAFS beamlines.

## 11. Results   

Although one of the main purposes of the first round robin on metal foils will be to establish the procedure for further tests with samples that are nearer to the typical user sample, we expect significant results from the comparison of the XAFS spectra measured at so many different sources. The discussion and interpretation of the results will be based on a detailed comparison of the differences between spectra, mainly of the raw data {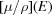 and of 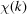}. The comparability of the energy scales, the spectral resolution but also of reproducibility (*e.g.* scan-to-scan energy variations) and obvious systematic errors and glitches are particular areas of inquiry.

Additionally there will be detailed comparison of EXAFS fit results and the influence of data quality (data range, noise level, *etc.*) on determined EXAFS parameters. The evaluation will be carried out by one person, preferably a designated doctoral student, to avoid artefacts caused by different use of the evaluation software. The question whether different software or different use of one software suite is causing differences in the extracted structural parameters is not within the scope of the proposed round robin. However, it is an equally interesting question and the measured spectra could be used for this purpose at a later stage.

The results of this enterprise will be published in strictly anonymized form and should not be understood as an attempt of ‘beamline ranking’. However, generalized recommendations, for instance about optimum sources for specific tasks or higher harmonics suppression schemes, are within the scope of the round robin results.

## Figures and Tables

**Figure 3:**
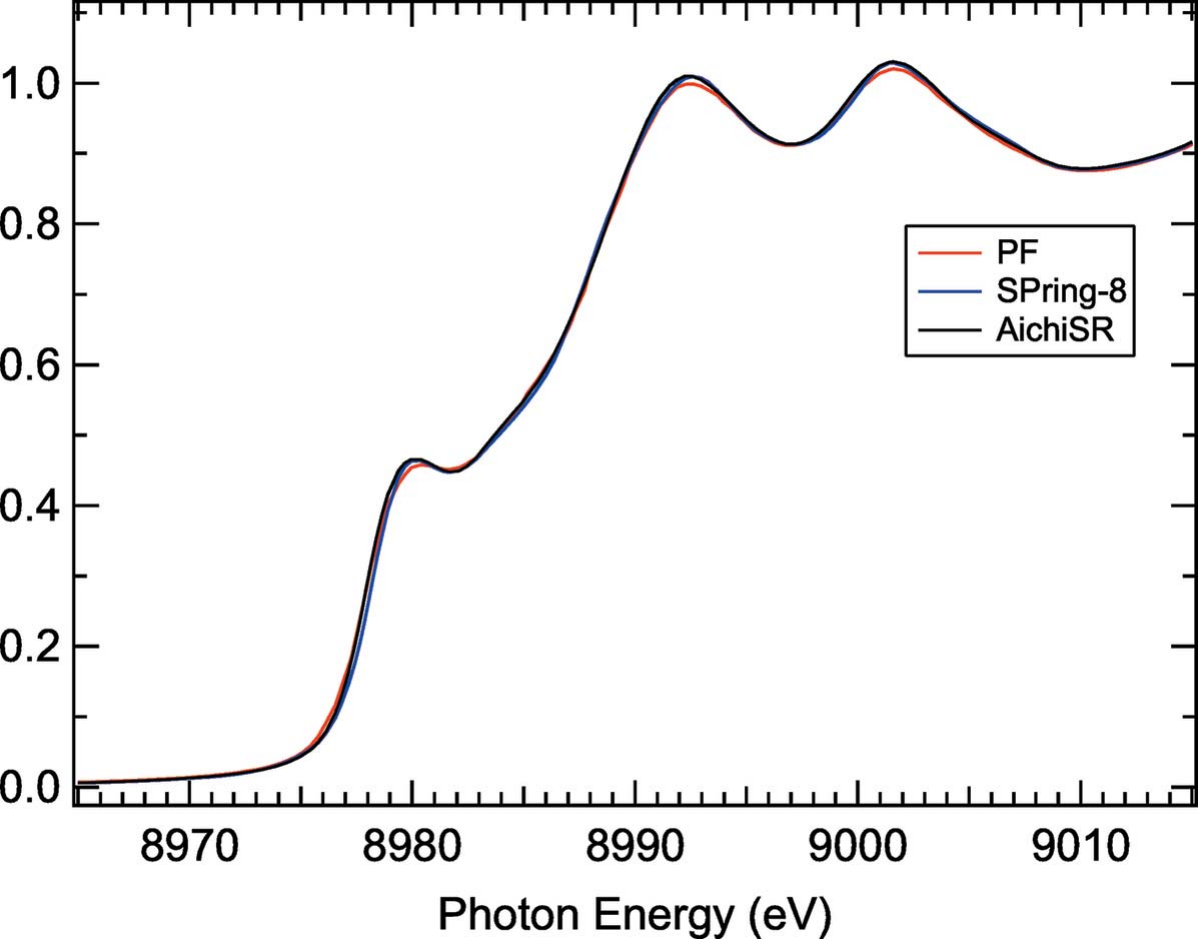
Cu foil XANES spectra measured at Photon Factory (red), SPring-8 (blue) and AichiSR (black). There are some differences largely because of slit widths before and after the double-crystal monochromators of each beamline. These data were obtained by the Japanese Round Robin activity (K. Kimijima and M. Masao).
